# Prevalence of hyposegmentation of granulocytes/Pelger-Huët anomaly in different canine breeds: a Bayesian approach

**DOI:** 10.3389/fvets.2025.1602474

**Published:** 2025-06-03

**Authors:** Erika Carli, Ester Salsecci, Roberta Calleo, Ileana Baldi

**Affiliations:** ^1^Unit of Biostatistics, Epidemiology and Public Health, Department of Cardiac, Thoracic, Vascular Sciences and Public Health, University of Padova, Padua, Italy; ^2^San Marco Veterinary Clinic and Laboratory, Padua, Italy

**Keywords:** Pelger-Huët anomaly, hyposegmentation, prevalence, Bayesian analysis, dog, Bayesian analysis reporting guidelines, BARG, PHA

## Abstract

Pelger-Huët anomaly (PHA) is a benign congenital hematological disorder first observed in humans and occasionally reported in dogs. It has been mainly described in Australian Shepherd Dog (ASD) with a prevalence of 9.8–13% where, based on a genetic study, it was recently renamed hyposegmentation of granulocytes (HG). Prevalences in other canine breeds have not been documented. This study aims to: (1) estimate the prevalence of HG/PHA across various breeds, (2) quantify the uncertainty of the estimated values using a Bayesian approach, and (3) identify affected breeds not previously documented. This cross-sectional study was based on the CBC database of the San Marco Veterinary Clinic and Laboratory (Padua, Italy) from 2001 to 2024. Data were collected from dogs diagnosed with HG/PHA and breeds previously reported as affected. To handle limited data and provide reliable estimates, Bayesian analysis was performed to estimate the prevalence of the anomaly and its uncertainty, using posterior probabilities from an informative prior model. The analysis adhered to the Bayesian Analysis Reporting Guidelines (BARG). The study included 5,716 dogs: German Shepherd (GS, 40%), Dachshund (DA, 22.6%), Cocker Spaniel (CS, 17.3%), Border Collie (BC, 9.5%), ASD (5.9%), Samoyed (SA, 2.2%), Boston Terrier (BT, 1.6%), Australian Cattle Dog (ACD, 0.7%) and Basenji (BA, 0.2%). Overall, HG/PHA was found in 0.45% dogs, specifically in ASD (7.1%), SA (0.8%) and DA (0.08%) and not in the other breeds. The prevalence estimates were 6.47% in ASD with 95% Credible Interval (95% CrI) from 4.22 to 9.18%, 0.32% (95% CrI: 0.04, 1.11%) in SA, 0.2% (95% CrI: 0.02, 0.86%) in BA, 0.18% (95% CrI: 0.02, 0.77%) in ACD, 0.16% (95% CrI: 0.02, 0.64%) in BT, 0.11% (95% CrI: 0.02, 0.30%) in DA, 0.10% (95% CrI: 0.01, 0.34%) in BC, 0.08% (95% CrI: 0.01, 0.25%) in CS, 0.05% (95% CrI: 0.01, 0.15%) in GS. HG/PHA was newly identified in DA. This study, using laboratory data collected over two decades and analyzed with Bayesian methods, could be considered representative of the prevalence of HG/PHA in multiple canine breeds. It is the first study to estimate the prevalence of HG/PHA beyond ASD, highlighting breed-specific differences in a real-world setting.

## 1 Introduction

The Pelger-Huët anomaly (PHA) is a benign congenital hematological disorder in which granulocytes (especially neutrophils) are characterized by hyposegmentation of nuclei and mature coarse chromatin patterns (1, 2). PHA was first described in humans (3, 4), where it is inherited as an autosomal dominant trait caused by mutations in the lamin B receptor (*LBR1*) gene (5, 6).

PHA has been sporadically described in domestic animals as rabbits (7), cats (8–10), horses (11, 12) and different canine breeds (13).

Traditionally, PHA has been considered an autosomal dominant trait in dogs (as in human medicine) and has often been reported as an asymptomatic heterozygous form. Recently, a homozygous *LMBR1L* splice site variant (which is therefore different from LBR1 in humans) has been identified as a possible causal variant in affected Australian Shepherd Dogs (ASD) (14). In this breed, the neutrophilic anomaly has also been shown to follow an autosomal recessive mode of inheritance (14). Therefore, it has been suggested that the anomaly observed in ASD may not be homologous to human PHA, and consequently the use of the term ‘hyposegmentation of granulocytes’ (HG) has been advised for the ASD condition, reserving the term PHA exclusively for the *LBR1*-related mutations anomaly (14).

The congenital form of PHA must be differentiated from pseudo-PHA, an acquired form in which similar morphological changes of white blood cells are associated with pathological conditions such as inflammation/infection (15, 16), some drug therapies (17), leukemia (16, 18), and myelodysplasia with asynchronous neutrophil maturation (16).

The anomaly has been reported primarily in ASD with frequencies ranging from 9.8% (1) to 13% (14). Historically, PHA has also been observed in other breeds such as Australian cattle dog (ACD), Basenji (BA), Border Collie (BC), Boston terrier (BT), Cocker spaniel (CS), Black and Tan and Redbone Coonhound, Danish/Swedish Farmdogs, Foxhounds, German Shepherd (GS), Samoyed (SA) (13) and in mixed breed dogs (19). However, for each of these breeds, limited evidence is still available and relies on case reports/case series (20–22) or anecdotical descriptions of single or related subjects. Probably due to the rarity of the anomaly, to the knowledge of the authors, no population study or prevalence and/or incidence of PHA have been documented in breeds other than ASD.

The Bayesian analysis permits to incorporate external, independent prior knowledge or belief from previous studies in the fitted model (23, 24), quantifying uncertainty about the magnitude of effect (23, 25) by computationally robust estimates and credible intervals (CrI) for derived parameter values (23, 26). The use of Bayesian statistics over frequentist estimation in a small sample size is commonly recommended (27). In a systematic review comparing Bayesian versus frequentist estimation with a small sample size, the authors encouraged making well-considered decisions about all priors, preferring a thoughtful prior instead of a naive one (28).

The aims of this study are (1) to estimate the overall prevalence of PHA in various canine breeds, (2) to use a Bayesian approach to quantify the uncertainty of the estimated values and (3) to describe, for the first time, breeds affected by PHA that have not been previously reported in the literature.

## 2 Materials and methods

### 2.1 Setting, data source, and definition

This study was carried out at the San Marco Veterinary Laboratory (Padua, Italy), which is the referral laboratory for patients of the San Marco Private Clinic (the same institute) and for patients of veterinarians scattered throughout Italy. All analysis reports have been electronically recorded and stored by POA System Plus 9.0® software to be available for consultation at any time. Among the complete blood count (CBC) recorded reports (*n* = 720,002), only those of dogs referred to the San Marco Veterinary Clinic (*n* = 170,747) had the essential anamnestic and clinical information to be considered eligible for the present study. As a laboratory rule, each CBC recorded in the database must include an automated analysis of the sample together with a microscopic evaluation of the peripheral blood smear (PBS).

The samples used in the present study were previously collected from clinicians during routine veterinary activity from healthy and unhealthy dogs. The samples were taken solely for diagnostic purposes with the owner’s informed consent.

As the genetic study of the anomaly was not a primary objective of this study and given that, to the knowledge of the authors, the genetic findings reported in ASD (14) have not been described in other breeds so far, the broader term HG/PHA will be used to identify the morphological characteristics of granulocytes traditionally reported as PHA in the dog.

### 2.2 Dogs and selection criteria

This cross-sectional study was conducted throughout the San Marco Veterinary Clinic and Laboratory CBC database in two steps during the period between 30 April 2001 and 24 February 2024.

An initial data extraction was performed, focusing on reports of patients with suspected or diagnosed HG/PHA, to identify and to investigate dogs potentially affected by the anomaly. To be considered suitable for the present study, the inclusion criteria were: (1) signalment with at least breed indication; (2) presence of at least 40% hyposegmented neutrophils with the typical appearance of HG/PHA. Exclusion criteria were: (1) no second evaluation when HG/PHA was suspected but not clearly diagnosed by PBS in the first presentation; (2) presence of myeloid neoplasia associated with HG/PHA; (3) presence of mild infection/inflammation attested by increased reactive C protein (>20 mg/L) (29), neutrophilic leukocytosis and with or without toxic cytoplasmic changes, in the absence of confirmation of the HG/PHA by other post-recovery CBCs.

Medical records including signalment, clinical and therapeutic history, physical examination, laboratory analysis (i.e., biochemical profile, serum protein electrophoresis, hemostatic profile, and urine analysis), imaging, and all other tests or procedures necessary to make the final diagnosis were evaluated to exclude causes of pseudo-PHA. When available, historical medical records were consulted to determine whether HG/PHA was confirmed by other CBC performed after diagnosis.

To obtain data from all dogs belonging to breeds eligible for the study to estimate breed-specific prevalences, a second data extraction was performed from the database, covering the same period. Dogs were enrolled based on the following inclusion criteria: (1) belonging to one of the breeds of dogs with HG/PHA previously identified (including both new breeds and those previously reported in literature); (2) belonging to one of the breeds reported in the literature as affected by HG/PHA (13); (3) having a CBC report.

Data were collected in separate groups according to breed, and the results of CBC and PBS microscopic evaluations were used as a proxy measure of the prevalence of HG/PHA. Breed-specific prevalences were subsequently estimated based on the available breeds.

### 2.3 Samples, laboratory analysis, and diagnosis

The K3EDTA whole blood samples were analyzed using automatic cells counters (ADVIA®120, ADVIA®2,120 and ADVIA®2120i Hematology System, Siemens Healthineers GmbH, Erlangen, Germany) within 1 h after collection. PBSs were prepared accurately immediately after collection (in 30′) and stained with a modified Wright stain using a hematology slide stainer (Aerospray®LB-S44, Delcon, Bergamo, Italy).

**Figure 1:**
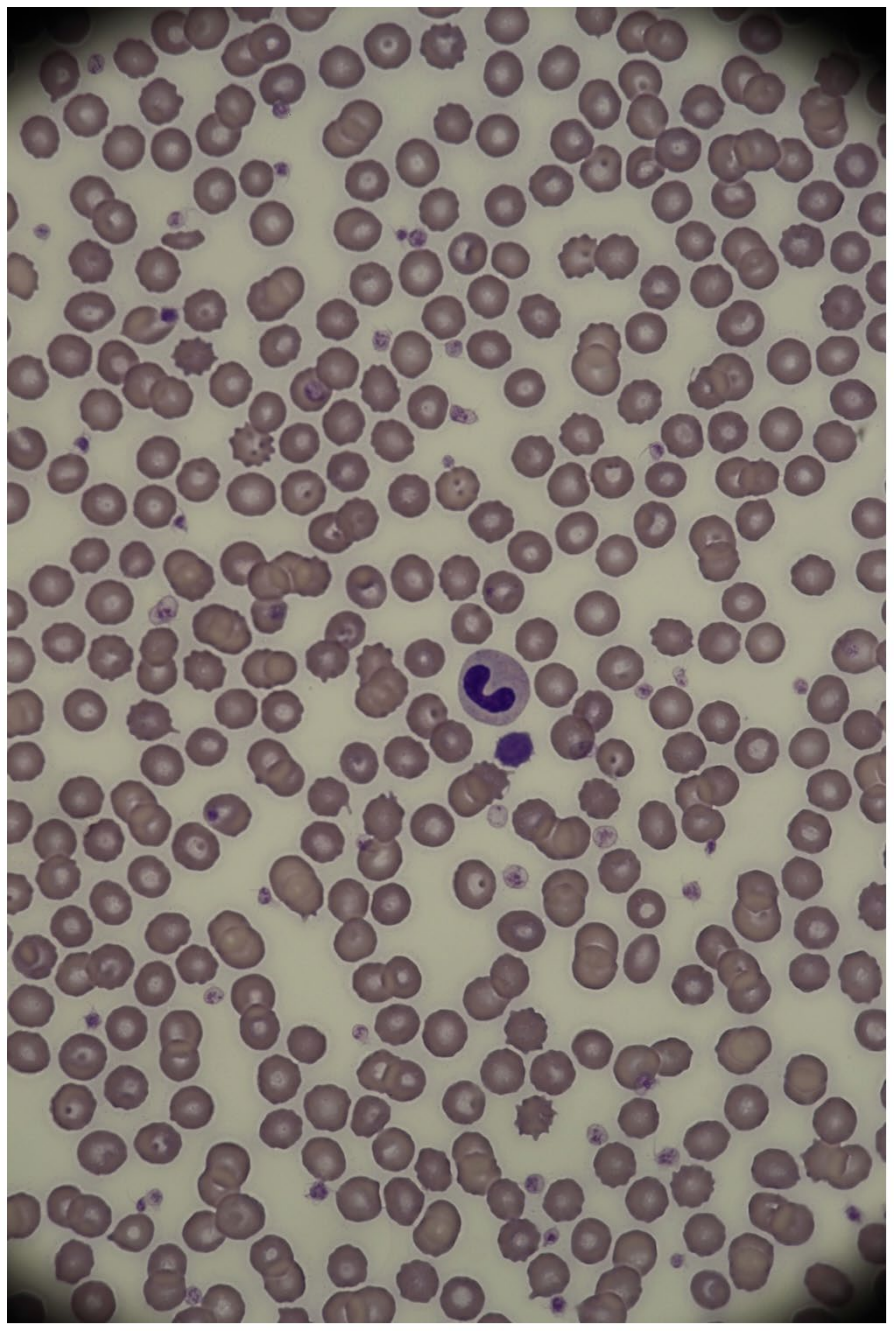
Neutrophil in an Australian shepherd dog affected by HG/PHA (1,000X, modified Wright stain). Courtesy of the San Marco Veterinary Laboratory (Padua, Italy).

The diagnosis was based on an optical microscopical examination of the blood smears with manual differential white blood cell counts by an experienced hematologist. Neutrophils with HG/PHA appearance frequently showed band- or metamyelocyte-shaped nuclei, with fully mature, coarse, densely clumped nuclear chromatin (Figure 1). Bilobed nuclei with ‘pince-nez’ or ‘dumbbell’ appearances or round to oval, nonlobulated or peanut-shaped nuclei could also be present (30). According to the inclusion criteria, only dogs that showed the typical morphological characteristics previously described, in most neutrophils and possibly eosinophils, were considered affected by HG/PHA.

### 2.4 Statistical analysis

#### 2.4.1 Descriptive analysis

The study population was analyzed according to breed and each breed was characterized by sex, sexual status, and age at diagnosis. Quantitative data were expressed as mean ± standard deviation, if normally distributed, and as median and interquartile range (IQR), if not normally distributed. Categorical variables were expressed as counts and percentages in each category.

The prevalence of HG/PHA in the different breeds was estimated and reported as a percentage.

#### 2.4.2 Bayesian analysis

**Figure 2:**
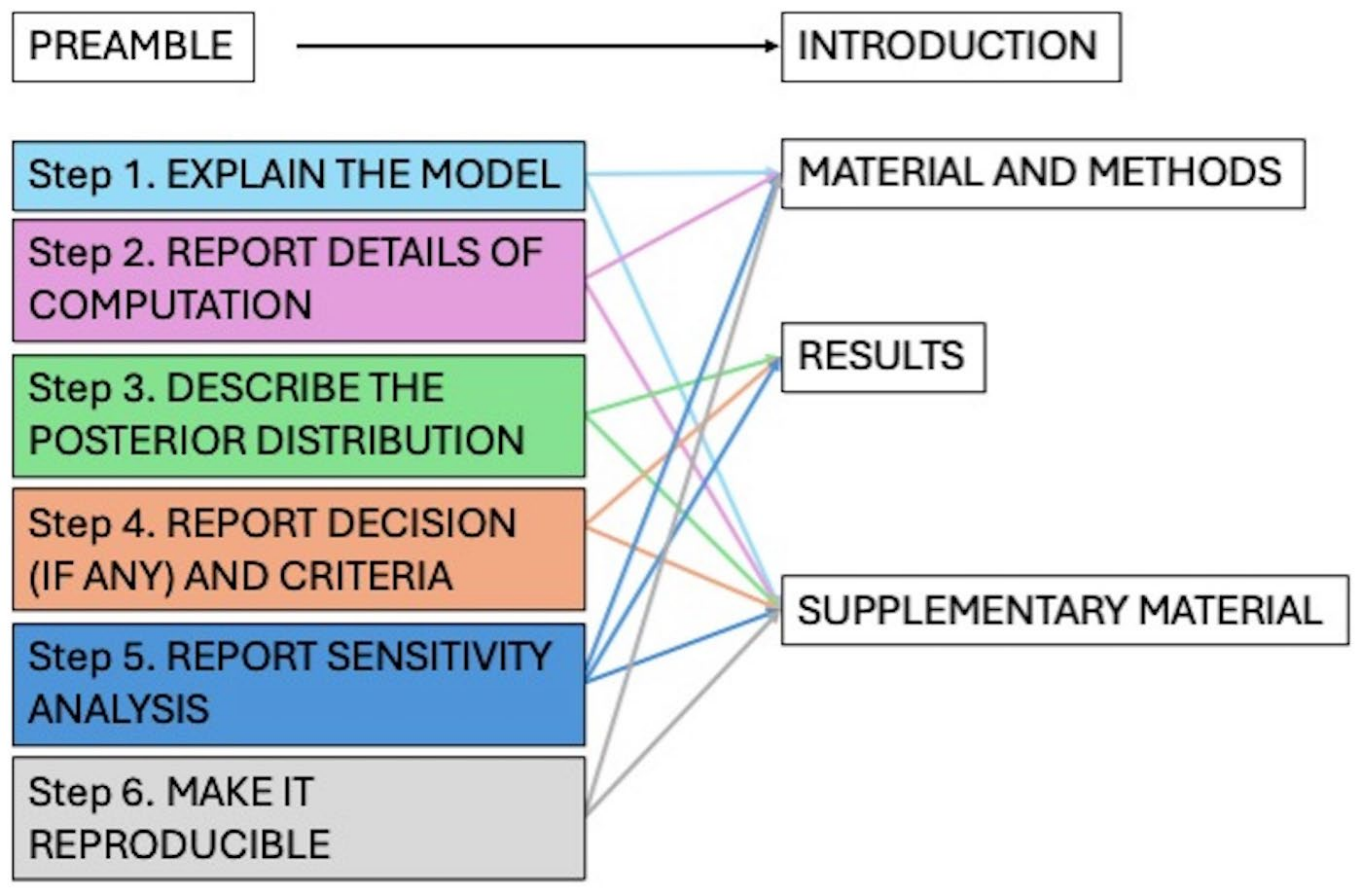
List of the items for the Bayesian analysis reported guidelines (BARG) and location in the paper.

To improve the quality, transparency, and reproducibility of the statistical approach, the Bayesian Analysis Reporting Guidelines (BARG) were used to conduct and report the present analysis. The guidelines propose following a list of key points including model explanation, details of computation, posterior distribution description, model comparison, and sensitivity analysis (26). To accurately reflect current knowledge, informative priors (InfPr) model was elicited and evaluated against a broad prior model and a mildly informative prior model through a sensitivity analysis. Figure 2 presents the list of items requested by the guidelines addressed throughout the manuscript. Detailed steps of the analysis can be consulted in the Supplementary material.

Statistical analysis was implemented using R^1^ version 4.4.1 (2024-06-14). Bayesian models were interfaced with Stan via the *brm* function in the ‘*brms*’ package (31), the graphic MCMC evaluation was implemented using ‘bayesplot’ package, and some parameters were obtained using ‘bayestestR’ and ‘tydibayes’ packages. Other packages used are ‘tydiverse’, ‘kableExtra’ and ‘ggplot2’.

## 3 Results

The dogs (*n* = 5,716) included in the study were GS (*n* = 2,286, 40%), DA (*n* = 1,293, 22.6%), CS (*n* = 990, 17.3%), BC (*n* = 541, 9.5%), ASD (*n* = 338, 5.9%), SA (*n* = 126, 2.2%), BT (*n* = 91, 1.6%), ACD (*n* = 41, 0.7%), and BA (*n* = 10, 0.2%). The dogs were 47.8% female and 52.2% male and ranged in age from 0.09 to 22.4 years, with a median value (IQR) of 6.47 (6.72) years. The demographic characteristics of the dogs are summarized in Table 1.

**Table 1:** Characteristics of the dogs at the baseline in the total study population and in the HG/PHA affected and not affected groups.

Variable	Overall dogs	HG/PHA	
		absent (*n* = 5,690)	present (*n* = 26)
Breed (*n* = 5,716)			
GS (%)	2,286 (40)	2,286 (40.2)	0
DA (%)	1,293 (22.6)	1,292 (22.7)	1 (3.8)
CS (%)	990 (17.3)	990 (17.4)	0
BD (%)	541 (9.5)	541 (9.5)	0
ASD (%)	338 (5.9)	314 (5.5)	24 (92.3)
SA (%)	126 (2.2)	125 (2.2)	1 (3.8)
BT (%)	91 (1.6)	91 (1.6)	0
ACD (%)	41 (0.7)	41 (0.7)	0
BA (%)	10 (0.2)	10 (0.2)	0
Sex (*n* = 5,716)			
Male (%)	2,983 (52.2)	2,968 (52.2)	15 (57.7)
Female (%)	2,733 (47.8)	2,722 (47.8)	11 (42.3)
Sexual status (*n* = 5,714)			
Intact (%)	3,624 (63.4)	3,608 (63.4)	16 (61.5)
Neutered (%)	2090 (36.6)	2080 (36.6)	10 (38.5)
Age, years (*n* = 5,716), median (IQR)	6.47 (6.72)	6.50 (6.71)	2.83 (4.27)

ACD, Australian cattle dog; ASD, Australian Shepherd Dog; BA, Basenji; BC, Border Collie; BT, Boston terrier; CS, Cocker spaniel; DA, Dachshund; GS, German Shepherd; IQR, Interquartile Range; SA, Samoyed.

Overall, HG/PHA was observed in 26 out of 5,716 (0.45%) dogs. Twenty-four cases were among ASD (7.1%), one SA (0.79%), one DA (0.08%) and absent in the other breeds. The percentage of pelgeroid neutrophils counted by microscopy evaluation was available in most affected dogs with a median value of 88% (IQR: 16%). In three cases of ASD, the percentages of typical neutrophils were not recorded at the time of evaluation and could not be recovered because blood smear slides were no longer available. The diagnosis was based on multiple CBC evaluation in 17/26 (65%) dogs. Dogs with a unique CBC were considered affected because they did not meet the exclusion criteria and belonged to breeds previously known to be affected by HG/PHA (13). Seven ASD dogs were excluded because they have a unique CBC in which the anomaly was associated with signs of inflammation (*n* = 4), or the diagnosis was uncertain (*n* = 3).

In the DA dog, the diagnosis was considered plausible in the evaluation of CBC with most neutrophils with the characteristics of HG/PHA and in the absence of an underlying disease or drug administration associated with pseudo-PHA development. The data of the offspring and siblings are not available due to the retrospective nature of the study.

The detailed results of the Bayesian analysis are reported in the Supplementary material. The InfPr model best fitted the data, capturing the trend of them. The estimated prevalences and 95%CrI of HG/PHA obtained for each breed were summarized in Table 2. Based on the high values of the posterior probability of direction (PD = 100%) and practical significance (PS = 1.00) obtained for each breed, the estimates appeared robust and strongly supported by the data. These results suggested a meaningful difference between the different breeds. A slightly positive prevalence was estimated for the male sex (see the Supplementary material), but it was moderately supported, with PD = 69.5%, PS = 0.52 and not statistically significant. Consequently, it is not clear whether male sex influences the prevalence of HG/PHA within breeds.

**Table 2:** Estimated prevalences of HG/PHA related to breed.

Breed	Estimated prevalence (%)	Lower 95%CrI (%)	Upper 95%CrI (%)
ASD	6.47	4.22	9.18
SA	0.32	0.04	1.11
BA	0.20	0.02	0.86
ACD	0.18	0.02	0.77
BT	0.16	0.02	0.64
DA	0.11	0.02	0.30
BC	0.10	0.01	0.34
CS	0.08	0.01	0.25
GS	0.05	0.01	0.15

Estimated prevalence (mean probability), Low 95%CrI and Upper 95%CrI (Equal Tailed Interval) were estimated by Bayesian analysis. ACD, Australian cattle dog; ASD, Australian Shepherd Dog; BA, Basenji; BC, Border Collie; BT, Boston terrier; 95%CrI, 95% Credible Interval; CS, Cocker spaniel; DA, Dachshund; GS, German Shepherd; SA, Samoyed.

## 4 Discussion

This study describes the prevalence of HG/PHA in various canine breeds using hematologic reports from a large laboratory database collected over a 20-year period. By analyzing data from routine laboratory procedures and from dogs evaluated for reasons unrelated to HG/PHA, our findings potentially reflect the true prevalence of this anomaly in a real-world setting.

The probabilistic nature of the Bayesian fitted model used in the present work allows for uncertainty quantifications in the estimated prevalences of HG/PHA based on breeds and provides clinicians with an idea of the frequency, but at the same time of the rarity, of this anomaly in the dog. By the Bayesian approach, what was already known by the parameter (the prior distribution) is combined with current evidence as support (the data we collect), resulting in updated knowledge of the parameter (posterior probability distributions) (27) that is clinically useful. The use of Bayesian over frequentist estimation in small sample size contexts, as in the present study, is recommended because it can be performed without losing power and retaining precision (27). Furthermore, since the construction of thoughtful priors is strongly encouraged in small sample size contexts (28), posterior probabilities from broad to informative three prior models were compared by sensitivity analysis, and the most informative one was selected to estimate the prevalence and uncertainty of the anomaly for the breeds included in this work. Furthermore, to produce rigorous, transparent, and reproducible analysis and to enhance the robustness and credibility of results, a workflow that adheres to the BARG steps (26) was applied.

For the first time, we report prevalences of HG/PHA in some canine breeds other than ASD. So far, only single cases or descriptions of related dogs have been reported for these breeds (13). Our results confirm that HG/PHA is a rare disease in ACD, BA, BC, BT, CS, GS, and SA with an estimated prevalence between 0.05–0.32%. Lower frequencies of 0.01 to 0.1% for human PHA were described. However, higher frequencies have historically been observed in clusters, such as in Sweden (0.6%) and Germany (1.01%) (30). In human hematological laboratory, only a part of the blood samples analyzed are manually reviewed by smear microscopic evaluation, for example, when it is suggested by a clinical or instrumental (i.e., ‘flags’ report) suspicious of disease (32, 33); consequently, this morphological anomaly may not be detected routinely. On the contrary, in veterinary medicine, microscopic evaluation of PBS is strongly encouraged in all patients (34), and in a reference high-quality laboratory such as the one where this work was carried out, it is routinely performed in both healthy and unhealthy patients. Thus, our results might approximate the true prevalence of HG/PHA in the breeds considered. It should be noted that a small number of subjects were included in the BA and ACD groups and more dogs must be evaluated to better estimate the real prevalences of the anomaly in these breeds.

We estimated a prevalence of HG/PHA of 6.47% in ASD, less than reported in the other two studies available in the literature conducted in the United States (1) and Switzerland (14). This result could reflect a geographical difference distribution of the anomaly, a different intensity of inbreeding, or a different line breeding in Italy compared to other parts of the world. In agreement, historically geographical clustering of human PHA has been reported (35–37). On the other hand, a slight underestimate of prevalence could not be excluded, as seven subjects with significant inflammatory conditions and uncertain diagnosis were not included in the study due to the absence of a confirmatory CBC performed after recovery. It would be interesting to apply our model to datasets obtained from other geographic areas to gain additional information on breed-specific frequencies and enable comparative analysis.

Interestingly, even if HG/PHA remains generally uncommon in dogs, our data estimated different prevalences between breeds: higher in ASD and lower in the others. Recently, a mutation in the LMBR1L gene (so different from LBR1 in humans) has been identified in affected ASD (14). It is unknown whether dogs of different breeds share the same mutation and the same inheritance pattern with ASD. On the other hand, it could not be excluded that in those breeds where HG/PHA is described with a lower prevalence, the anomaly may be caused by other genetic mutations or by the same genetic mutations that cause human PHA. However, our results could simply reflect a different real breed-related prevalence of the same genetic anomaly. Future genetic studies should clarify the mutation underlying the anomaly in these canine breeds.

Notably, we also documented HG/PHA (apparently congenital) in DA for the first time, suggesting that this breed could plausibly be added to the list of breeds affected by the anomaly.

A limitation of this study could be that not only healthy dogs are included due to the nature of the database used. The HG/PHA affected dogs reported in the present study were incidentally found during routine microscopic laboratory activity; therefore, the PBSs of the sick dogs were also evaluated. Restrictive inclusion criteria and careful evaluation of clinical records were adopted to minimize or exclude pseudo-PHA misclassification, particularly in cases where hematologic follow-up was unavailable, thus preventing overestimation. However, due to the retrospective nature of data collection, the restrictive inclusion criteria, and the lack of hematologic follow-up after recovery, also a slight underestimate of the prevalence of the anomaly cannot be ruled out.

Given the retrospective design of this work, another limitation is the inability to confirm whether there was a familial relationship between ASD affected by the anomaly that could potentially bias the assessment of its true prevalence. Furthermore, considering the limited presence of these breeds in Italy, there was a low number of dogs available to estimate the prevalence of HG/PHA for BA and ACD.

In conclusion, this real-world study, based on extensive data, provides valuable information on the prevalence of HG/PHA in nine canine breeds. For the first time, it reports prevalence estimates in breeds beyond ASD using reliable estimates obtained through a Bayesian approach and identifies DA as a newly affected breed. These findings enhance clinicians’ understanding on the frequency, but at the same time of the rarity, of HG/PHA in dogs, highlighting breed-specific differences. This study improves the epidemiological characterization of the anomaly, supporting potential applications in clinical practice, genetic research, and dog breeding practices.

## Data Availability

The data analyzed in this study is subject to the following licenses/restrictions: the raw data supporting the conclusions of this article will be made available by the authors upon request. Requests to access these datasets should be directed to erika.carli@sanmarcovet.it.

## Footnotes

^1^ https://www.r-project.org/

## References

[1] Latimer KS Campagnoli RP Danilenko DM. Pelger-Huët anomaly in Australian shepherds: 87 cases (1991-1997). Comp Haematol Int. (2000) 10:9–13.

[2] Colella R Hollensead SC. Understanding and recognizing the Pelger-Huët anomaly. Am J Clin Pathol. (2012) 137:358–66. doi: 10.1309/AJCP3G8MDUXYSCID, PMID: 22338047

[3] Pelger K. Demonstratie van een paar zeldzaam voorkomende typen van bloedlichaampjes en bespreking der patiënten. Ned Tijdschr Geneeskd. (1928) 72:1178.

[4] Huët GJ. Uber eine bisher unbekannte familiare anomalie der leukocyten. Klin Wochenschr. (1932) 2:1264–6.

[5] Hoffmann K Dreger CK Olins AL Olins DE Shultz LD Lucke B. Mutations in the gene encoding the Lamin B receptor produce an altered nuclear morphology in granulocytes (Pelger-Huët anomaly). Nat Genet. (2002) 31:410–4. doi: 10.1038/ng925, PMID: 12118250

[6] Hoffmann K Sperling K Olins AL Olins DE. The granulocyte nucleus and Lamin B receptor: avoiding the ovoid. Chromosoma. (2007) 116:227–35. doi: 10.1007/s00412-007-0094-8, PMID: 17245605

[7] Nachtsheim H. The Pelger-anomaly in man and rabbit; a mendelian character of the nuclei of the leucocytes. J Hered. (1950) 41:131–7. 15436969 10.1093/oxfordjournals.jhered.a106108

[8] Deshuillers P Raskin R Messick J. Pelger-Huët anomaly in a cat. Vet Clin Pathol. (2014) 43:337–41. doi: 10.1111/vcp.12176, PMID: 25115222

[9] Latimer KS Rakich PM Thompson DF. Pelger-Huët anomaly in cats. Vet Pathol. (1985) 22:370–4. 4035941 10.1177/030098588502200412

[10] Latimer KS Rowland GN Mahaffey MB. Homozygous Pelger-Huët anomaly and chondrodysplasia in a stillborn kitten. Vet Pathol. (1988) 25:325–8. 3407106 10.1177/030098588802500416

[11] Gill AF Gaunt S Sirninger J. Congenital Pelger-Huët anomaly in a horse. Vet Clin Pathol. (2006) 35:460–2. doi: 10.1111/j.1939-165x.2006.tb00165.x, PMID: 17123255

[12] Grondin TM DeWitt SF Keeton KS. Pelger-Huët anomaly in an Arabian horse. Vet Clin Pathol. (2007) 36:306–10. doi: 10.1111/j.1939-165x.2007.tb00231.x, PMID: 17806084

[13] Safra N Bannasch D. Genetic evaluation of inherited hematologic diseases In: Brooks MB Harr KE Seelig DM Wardrop KJ Weiss DJ, editors. Schalm's veterinary hematology. 7th ed. Hoboken: John Wiley & Sons, Inc (2022). 1337–50.

[14] Lourdes Frehner B Christen M Reichler IM Jagannathan V Novacco M Riond B. Autosomal recessive hyposegmentation of granulocytes in Australian shepherd dogs indicates a role for LMBR1L in myeloid leukocytes. PLoS Genet. (2023) 19:e1010805. doi: 10.1371/journal.pgen.1010805, PMID: 37347778 PMC10321630

[15] Shull RM Powell D. Acquired hyposegmentation of granulocytes (pseudo-Pelger-Huët anomaly) in a dog. Cornell Vet. (1979) 69:241–7. 477323

[16] Comazzi S Aresu L Weiss DJ. Neutrophil function disorders In: Brooks MB Harr KE Seelig DM Wardrop KJ Weiss DJ, editors. Schalm's veterinary hematology. 7th ed. Hoboken: John Wiley & Sons, Inc (2022). 347–53.

[17] Harvey JW. Evaluation of leukocytic disorders In: Harvey JW, editor. Veterinary hematology: A diagnostic guide and color atlas. 1st ed. Missouri: Elsevier Inc (2012). 122–76.

[18] Martínez-Caro J Agulla B Viñeta C Roura X Mesalles M Pastor J. Presumed pseudo-Pelger-Huët anomaly and basophilia secondary to chronic lymphocytic leukemia in a dog. Vet Clin Pathol. (2024) 53:202–8. doi: 10.1111/vcp.13347, PMID: 38622430

[19] Vale AM Tomaz LR Sousa RS Soto-Blanco B. Pelger-Huët anomaly in two related mixed-breed dogs. J Vet Diagn Invest. (2011) 23:863–5. doi: 10.1177/1040638711407891, PMID: 21908340

[20] Bowles CA Alsaker RD Wolfle TL. Studies of the Pelger-Huët anomaly in foxhounds. Am J Pathol. (1979) 96:237–48. 464021 PMC2042355

[21] Latimer KS Duncan JR Kircher IM. Nuclear segmentation, ultrastructure, and cytochemistry of blood cells from dogs with Pelger-Huët anomaly. J Comp Pathol. (1987) 97:61–72. 2435771 10.1016/0021-9975(87)90128-9

[22] Lukaszewska J Allison RW Stepkowska J. Congenital Pelger-Huët anomaly in a Danish/Swedish Farmdog: case report. Acta Vet Scand. (2011) 53:14–8. doi: 10.1186/1751-0147-53-14, PMID: 21362186 PMC3059283

[23] Vasishth S Nicenboim B Beckman ME Li F Kong EJ. Bayesian data analysis in the phonetic sciences: a tutorial introduction. J Phon. (2018) 71:147–61. doi: 10.1016/j.wocn.2018.07.008, PMID: 30197458 PMC6124675

[24] van de Schoot R Depaoli S King R Kramer B Märtens TMG Vannucci M. Bayesian statistics and modelling. Nat Rev Methods Primers. (2021) 1:1–25. doi: 10.1038/s43586-020-00003-0

[25] Woodward AP. Bayesian estimation in veterinary pharmacology: a conceptual and practical introduction. J Vet Pharmacol Ther. (2024) 47:322–52. doi: 10.1111/jvp.13433, PMID: 38385655

[26] Kruschke JK. Bayesian analysis reporting guidelines. Nat Hum Behav. (2021) 5:1282–91. doi: 10.1038/s41562-021-01177-7, PMID: 34400814 PMC8526359

[27] van de Schoot R Broere JJ Perryck KH Zondervan-Zwijnenburg M van Loey NE. Analyzing small data sets using Bayesian estimation: the case of posttraumatic stress symptoms following mechanical ventilation in burn survivors. Eur J Psychotraumatol. (2015) 6:25216. doi: 10.3402/ejpt.v6.25216, PMID: 25765534 PMC4357639

[28] Smid SC McNeish D Miočević M van de Schoot R. Bayesian versus frequentist estimation for structural equation models in small sample contexts: a systematic review. Struct Equ Model Multidiscip J. (2020) 27:131–61. doi: 10.1080/10705511.2019.1577140

[29] Ceron JJ Martinez-Subiela S Tecles F Caldin M. Acute phase proteins in diagnostics: more than expected. J Hell Vet Med Soc. (2017) 65:197–204. doi: 10.12681/jhvms.15535

[30] Speeckaert MM Verhelst C Koch A Speeckaert R Lacquet F. Pelger-Huët anomaly: a critical review of the literature. Acta Haematol. (2009) 121:202–6. doi: 10.1159/000220333, PMID: 19468205

[31] Bürkner PC. Brms: an R package for Bayesian multilevel models using stan. J Stat Softw. (2017) 80:1–28. doi: 10.18637/jss.v080.i01

[32] Gulati G Song J Florea AD Gong J. Purpose and criteria for blood smear scan, blood smear examination, and blood smear review. Ann Lab Med. (2013) 33:1–7. doi: 10.3343/alm.2013.33.1.1, PMID: 23301216 PMC3535191

[33] Winther-Larsen A Vestergaard EM Abildgaard A. Clinical value of smear review of flagged samples analyzed with the Sysmex XN hematology analyzer. Clin Chem Lab Med. (2024) 63:636–44. doi: 10.1515/cclm-2024-0973, PMID: 39321044

[34] Zitzer NC. The greatness of glass: importance of blood smear evaluation. Vet Clin North Am Small Anim Pract. (2023) 53:29–52. doi: 10.1016/j.cvsm.2022.07.005, PMID: 36400473

[35] Davidson WM Lawler SD Ackerley AG. The Pelger-Huët anomaly: investigation of family. A Ann Hum Genet. (1954) 19:1–9. 13208022 10.1111/j.1469-1809.1954.tb01259.x

[36] Rioux E St-Ameault G Brosseau C. The Pelger-Huët anomaly of leukocytes: description of a Quebec kindred. Can Med Assoc J. (1968) 99:621–4. 5686316 PMC1945286

[37] Milanesi B. L'anomalia nucleare leucocitaria di Pelger-Huët: studio morfologico, citochimico, citogenetico ed ultrastrutturale di 39 casi [the Pelger-Huët leukocyte nuclear anomaly: a morphologic, cytochemical, cytogenetic and ultrastructural study of 39 cases (authors' transl)]. Quad Sclavo Diagn. (1980) 15:1097–115. 7454963

