# Supplementary material for: Prevalence of hyposegmentation of granulocytes/Pelger-Huët anomaly in different canine breeds: a Bayesian approach
Source: Front Vet Sci. 2025 Jun 3;12:1602474. doi: 10.3389/fvets.2025.1602474 (PMC12188539; doi:10.3389/fvets.2025.1602474)
Supplement: Supplementary file 1 [file Data_Sheet_1.PDF]

SUPPLEMENTARY MATERIAL

The details of the following statistical analysis were reported using the same step numbers and letters as in the Bayesian Analysis Reporting Guidelines (BARG) table (Kruschke, 2021), ensuring clear correspondence between the text and the applied steps for an easy identification.

[1.A.] The dependent variable is the dichotomous variable presence/absence of HG/PHA and the predictors is the breed, a categorical variable in nine levels (ACD, ASD, BA, BC, BT, CS, DA, GS, SA).

[1.C.] The selection of the prior distribution for the prevalence of HG/PHA was based on available literature that suggests a frequency between 9.8% (Latimer et al., 2000) and 13% (Lourdes Frehner et al., 2023) for ASD, while no frequencies have been reported for the other breeds where the anomaly is rarely detected. On this basis, the authors hypothesized low prevalences for all breeds (negative coefficients) with a much lower prevalence in breeds other than ASD. Therefore, to best represent current knowledge, informative priors (InfPr) elicited as a normal distribution with a higher negative mean and a higher variance for ASD compared to the other breeds were specified. They were defined as β_0 (Intercept, ASD breed) ~ N (-2,3) and β_i (each other breed) ~ N (-4,1).

[1. B.] As the outcome variable HG/PHA is binary, the logistic link function is used that leaded to a model with the following general form:

$$logit(P(Yi=1))=\beta_0+\beta_1 \cdot Breed_{2i}+\beta_2 \cdot Breed_{3i}+\beta_3 \cdot Breed_{4i}+\beta_4 \cdot Breed_{5i}+......+\beta_7 \cdot Breed_{8i}$$

where $P(Yi=1)$*: Probability of an anomaly occurring for observation i.*

β_0*: Intercept, representing the log-odds of an anomaly for* $Breed_1$*.*

β_i*: Coefficients representing the effect of each breed on the reference breed*

$\beta_0 \sim Normal(-2,3)$

$\beta_k \sim Normal(-4,1)$ *for* $k=1,...,7$

[1.D.] The model was specified in the 'brms' syntax as:

```
brm(outcome ~ group,
    data = data,
    family = bernoulli(link = "logit"),
    prior= priors_fit,
    chains = 4,
    iter = 10000,
    seed = 092024)
```

[1.E.] A prior predictive check was reported to assess whether the chosen priors were consistent with the trends assumed by prior knowledge. After the prior predictive checking, the data and the intended distributions were well represented by all the priors (Figure 1 and Figure 2).

Figure 1. Prior predictive check

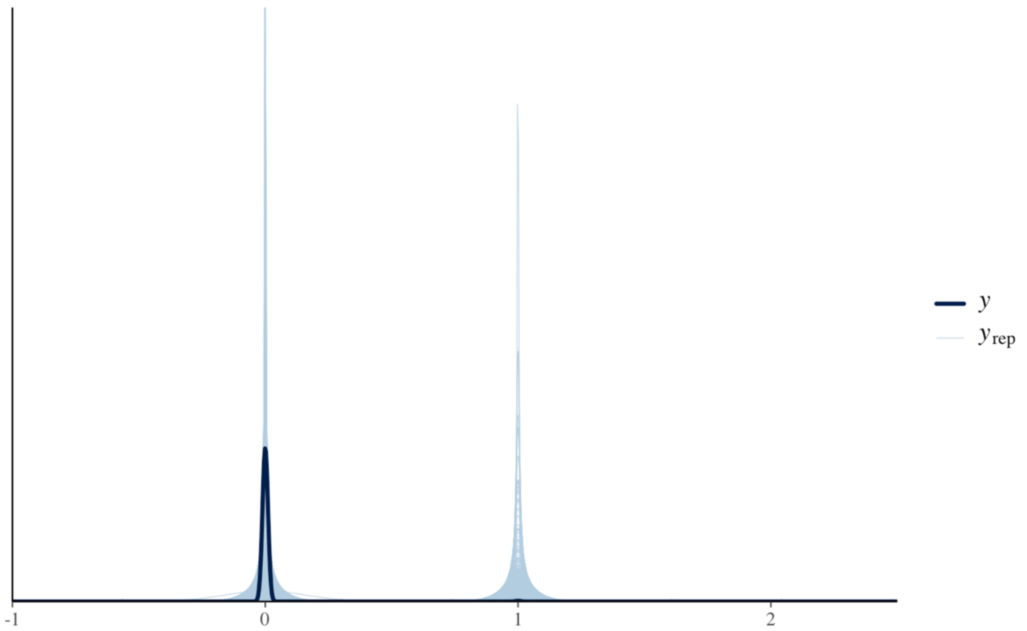

The observed data (y, dark blue) were compared with predictions (y_{rep}, light blue) generated by priors only.

Figure 2. Probability of HG/PHA sampled from the prior distribution compared to observed data across all breeds.

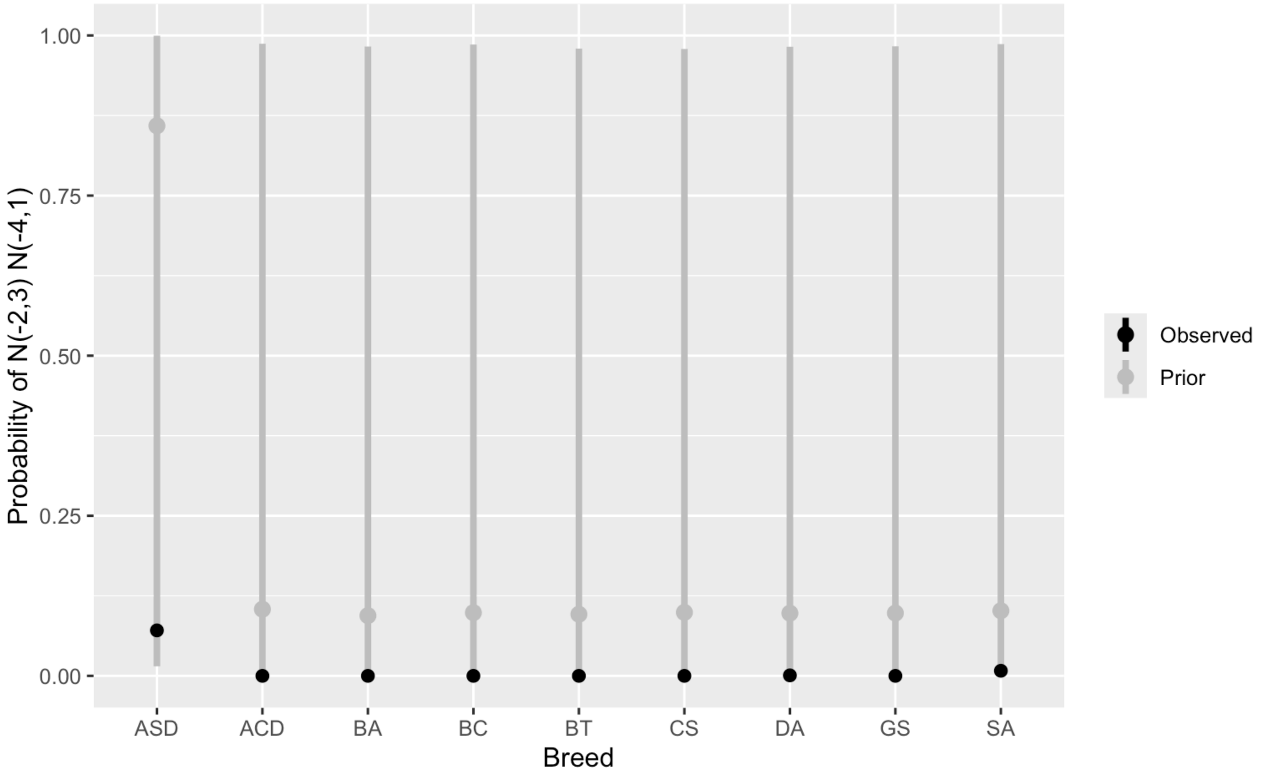

The prior predictions of the model are superimposed on the data as the probability of HG/PHA. The posterior predictive means are shown by the circles, and the error bars show the 95% credible intervals. ACD: Australian cattle dog; ASD: Australian Shepherd Dog; BA: Basenji; BC: Border Collie; BT: Boston terrier; CS: Cocker spaniel; DA: Dachshund; GS: German Shepherd; SA: Samoyed.

[2.B and 2.C]. For parameter estimation, the Markov Chain Monte Carlo (MCMC) simulations were employed: four chains, with 5,000 iterations for warm-up, each with 10,000 iterations, and with 20,000 total post-warm-up draws were used to ensure convergence. To check that the MCMC chains for every parameter have converged and are long enough to represent the distribution, the potential scale reduction factor (PSRF or R-hat) and the effective sample size (ESS) were obtained, respectively. An R-hat lower than 1.05 suggested that the chains converge to the same distribution, and an ESS >=10,000 suggested that they are long enough to have stable parameter estimates (Kruschke, 2021).

Furthermore, the mixing of chains by trace plots (Figure 3) and the autocorrelation plots were visually examined.

Figure 3. Trace plot of MCMC chains to evaluate convergence and mixing.

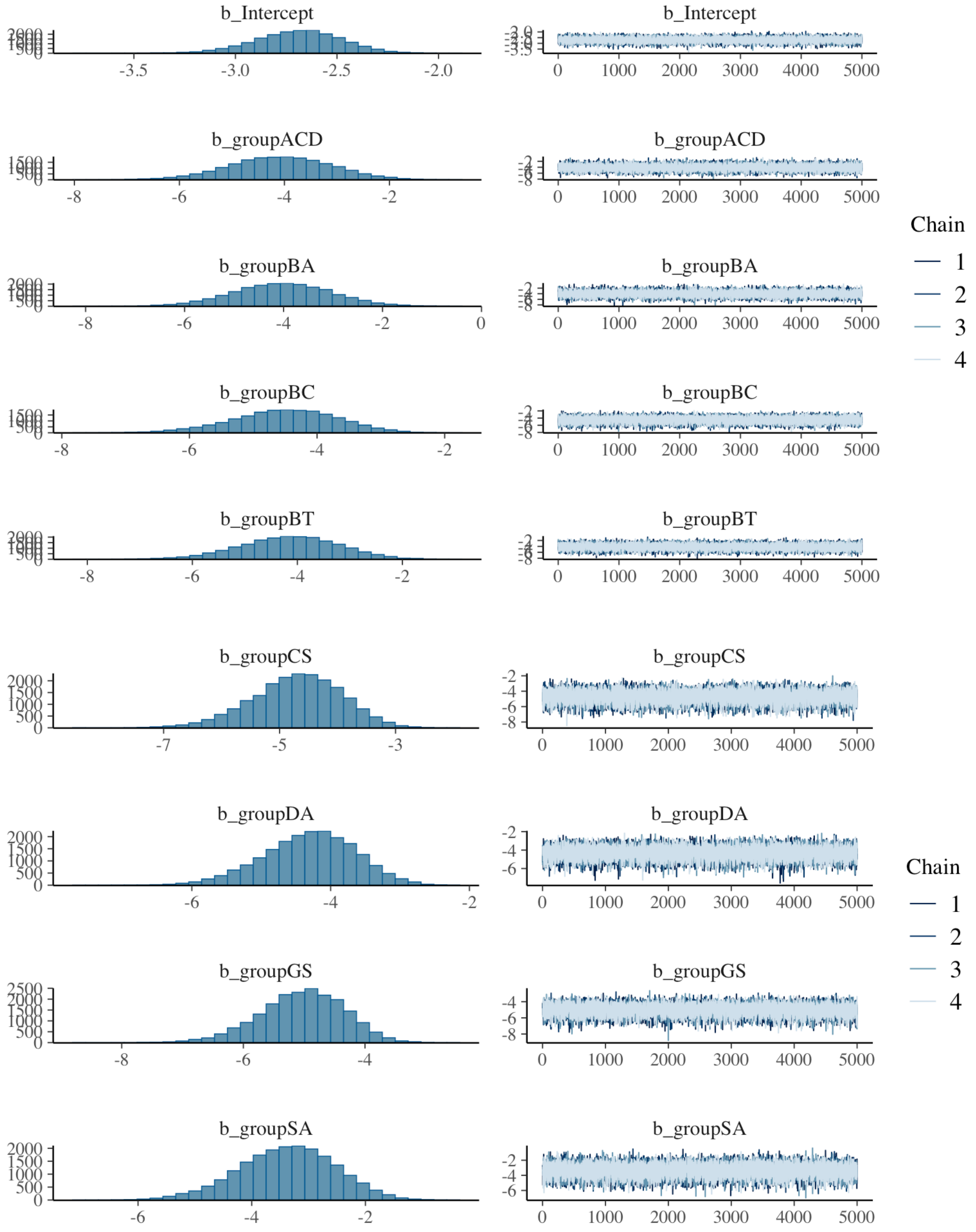

[3.A.] To check whether the simulated data from the model mimic the observed data, a posterior predictive check was graphically evaluated (Figure 4). The model has captured the trends of the data as shown in Figure 5.

Figure 4. Posterior predictive check

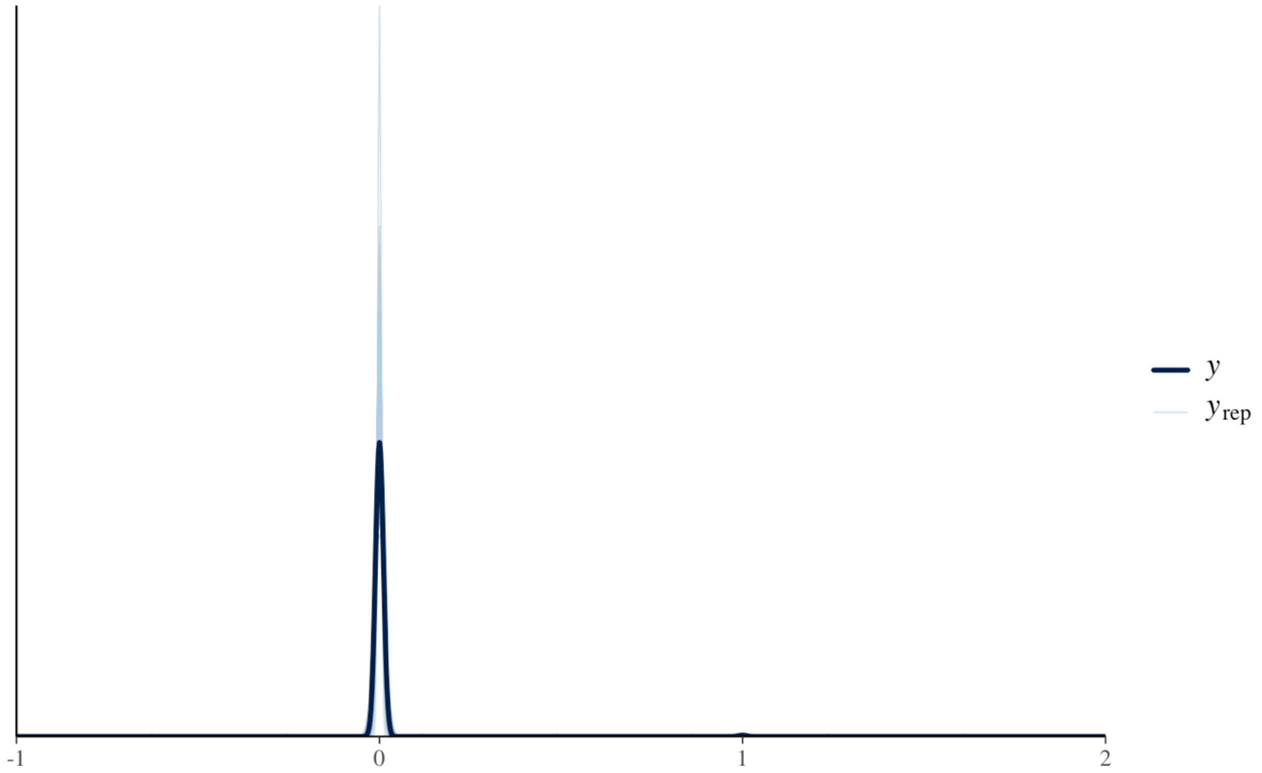

The observed data (y, dark blue) were compared with the simulated posterior predictive distribution (y_{rep}, light blue) after fitting model.

Figure 5. Probability of HG/PHA sampled from the posterior distribution compared to observed data across all breeds.

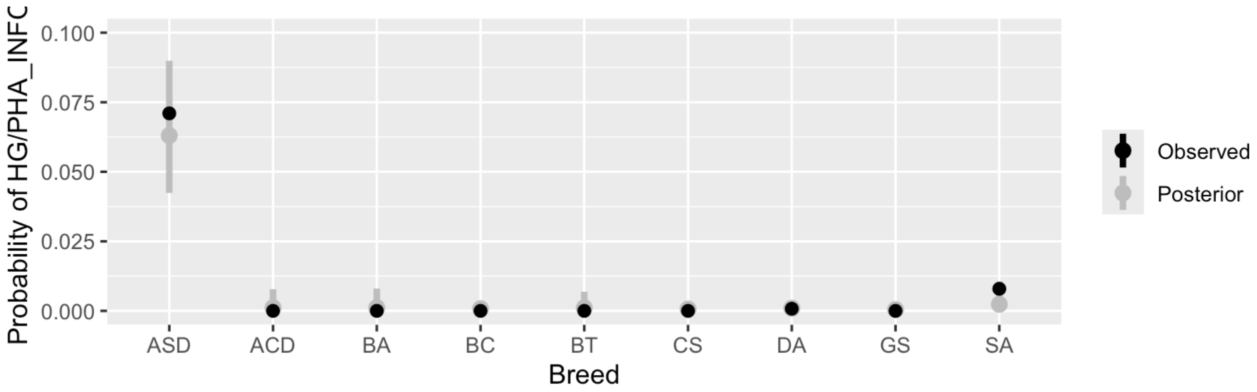

The posterior predictions of the model are superimposed on the data as the probability of HG/PHA. The posterior predictive means are shown by the circles, and the error bars show the 95% credible intervals. ACD: Australian cattle dog; ASD: Australian Shepherd Dog; BA: Basenji; BC: Border Collie; BT: Boston terrier; CS: Cocker spaniel; DA: Dachshund; GS: German Shepherd; SA: Samoyed.

The marginal posterior distributions of the parameters are unimodal and only slightly skewed.

[3. B.] The probabilities of HG/PHA obtained by means and 95% Equal Tailed Interval (ETI) for each breed are summarized in Table 2 in the result section of the paper.

[3. C.] To confirm the robustness of the analysis, the Bayes factor (BF) was considered to compare the models and the calculation of the central tendencies and limits of 95% CrI, and the posterior probability direction (PD) was considered to estimate the uncertainty of the value of the estimated parameters and to indicate the certainty of the estimated directionality of the effect. The parameter estimates for the breeds were strongly supported with PD=100% and PS=1 and statistically significant effects were obtained for each level of the breed variable. These results suggested a meaningful differences among the groups.

[5.B.] To evaluate how the model is sensitive to the choice of a prior distribution when using different priors, a sensitivity analysis was performed considering priors with a lower level of information than InfPr. Therefore, two other different priors were placed on. Broad priors (BrPr) were specified to be symmetric, to provide an opportunity for extreme responses, and to have limited influence on the results, while mildly informative priors (MildInfPr) mostly reflected the hypothesis of a lower estimated probability of HG/PA in breed other than ASD with a higher uncertainty with respect to

InfBr. A model with BrPr [β_0 (Intercept, ASD) ~ N (0,10) and for β_i (other breeds) ~ N (0,5)] and a model with MildInfPr [β_0 (Intercept, ASD) ~ N (-1,5) and for β_i (other breeds)] and with a logistic link function, with 12,000-15,000 iterations to reach 10,000 ESS were fitted. Prior predictive checks, MCMC convergences, and posterior predictive checks were evaluated as previously reported for the InfPr model.

[5.D and 4.D] The models were compared based on leave-one-out cross-validation (LOO) and BF to find the best fit. PD and PS were also evaluated, and the parameter estimates for the breeds were strongly supported by all models. The posterior predictive check shows that the InfPr model provides a better description of the data, with a better fit by the informative prior elicited. Based on the LOO model comparison, the InfPr model performs similarly to the other models. However, the BF comparisons favour the InfPr model, with evidence supporting it over both MildPr (BF=56.47) and BrPr (717.19). Also, PD and PS are slightly better for InfPr. When the summary tables and graphs of the posterior distributions are inspected, the posterior estimates of the parameters of the MildInfPr and InfPr models appear similar to those obtained from the BrPr model. However, the posterior estimates for the uncertainty (width) of the distributions were lower for the InfPr model. For these reasons, the InfPr model was considered the best choice.

A model (InfSexPr) with the same specification of the InfPr model, but also including sex, a categorical variable in two levels (M/F), was fitted and evaluated as previously reported for the other models. The prior for the variable sex was defined as ~ N (0, 2). A slightly positive parameter estimate for the male sex was observed, but it is moderately supported, with PD=69.5%, PS=0.52, and it is not statistically significant. Consequently, it is not clear whether male sex influences the prevalence of HG/PHA within breeds. The InfPr model shows performance comparable to that of the InfSexPr model, with a slightly stronger preference indicated by BF = 4.42. Given its simplicity and similar predictive accuracy, the InfPr model is preferred due to its parsimony and efficiency.

References

Latimer KS, Campagnoli RP, Danilenko DM. Pelger–Huët anomaly in Australian shepherds: 87 cases (1991–1997). *Comp Haematol Intern.* (2000) 10: 9-13.

Lourdes Frehner B, Christen M, Reichler IM, Jagannathan V, Novacco M, Riond B, Peters LM, Suárez Sánchez-Andrade J, Pieńkowska-Schelling A, Schelling C, Kipar A, Leeb T, Balogh O. Autosomal recessive hyposegmentation of granulocytes in Australian Shepherd Dogs indicates a role for LMBR1L in myeloid leukocytes. *PLoS Genet*. (2023) 19(6): e1010805. doi: 10.1371/journal.pgen.1010805.

Kruschke JK. Bayesian Analysis Reporting Guidelines. *Nat Hum Behav*. (2021) 5(10): 1282-91. doi: 10.1038/s41562-021-01177-7.
